# Crossover from two-frequency pulse compounds to escaping solitons

**DOI:** 10.1038/s41598-021-90705-6

**Published:** 2021-05-27

**Authors:** O. Melchert, S. Willms, U. Morgner, I. Babushkin, A. Demircan

**Affiliations:** 1 Institute of Quantum Optics, Leibniz Universität Hannover, Welfengarten 1, 30167 Hannover, Germany; 2 Cluster of Excellence PhoenixD, Welfengarten 1, 30167 Hannover, Germany; 3 Hannover Centre for Optical Technologies, Nienburger Str. 17, 30167 Hannover, Germany

**Keywords:** Nonlinear optics, Solitons

## Abstract

The nonlinear interaction of copropagating optical solitons enables a large variety of intriguing bound-states of light. We here investigate the interaction dynamics of two initially superimposed fundamental solitons at distinctly different frequencies. Both pulses are located in distinct domains of anomalous dispersion, separated by an interjacent domain of normal dispersion, so that group velocity matching can be achieved despite a vast frequency gap. We demonstrate the existence of two regions with different dynamical behavior. For small velocity mismatch we observe a domain in which a single heteronuclear pulse compound is formed, which is distinct from the usual concept of soliton molecules. The binding mechanism is realized by the mutual cross phase modulation of the interacting pulses. For large velocity mismatch both pulses escape their mutual binding and move away from each other. The crossover phase between these two cases exhibits two localized states with different velocity, consisting of a strong trapping pulse and weak trapped pulse. We detail a simplified theoretical approach which accurately estimates the parameter range in which compound states are formed. This trapping-to-escape transition allows to study the limits of pulse-bonding as a fundamental phenomenon in nonlinear optics, opening up new perspectives for the all-optical manipulation of light by light.

## Introduction

The nonlinear Schrödinger equation (NSE) constitutes a paradigmatic model in nonlinear optics that exhibits solitons, i.e. particle-like field solutions that exist due to a balance of dispersive and nonlinear effects^1–3^. Individual NSE solitons propagate without changing their shape and collisions between two such solitons do not affect their individual properties^4^. A characteristic of NSE solitons is the hyperbolic-secant shape, i.e. $\text{sech}$–shape, of their field envelope. The NSE solitons defining parameters involve the fiber parameters but a free parameter, given by the soliton duration or amplitude, is retained, allowing to define the pulse characteristics. If the NSE is perturbed by higher orders of dispersion, phase-matching effects can allow for the resonant generation of radiation^5–7^. In such a case, a soliton will suffer energy loss upon propagation. Hence, for NSE-type equations with more general dispersion relations, true solitons are not implied. However, for the particular case of anomalous second-order dispersion (2OD), vanishing third-order dispersion (3OD), and *positive* fourth-order dispersion (4OD), an exact soliton solution of $\text{sech}\times \text{tanh}$–shape exist^8^. In contrast to a NSE soliton, the properties of this “fixed-paramter” soliton solution are fully determined by the fiber parameters. Further, for the case of anomalous 2OD, vanishing 3OD, and *negative* 4OD, an exact fixed-parameter soliton solution of $\text{sech}^2$–shape was specified, its interaction dynamics studied, and a continuous family of solutions was shown to exist^9,10^. For a variant in which the propagation equation is governed by negative 4OD only, “pure-quartic solitons” where reported^11^. Recently, an exact $\text{sech}^2$–shaped fixed-parameter soliton solution for the case of anomalous 2OD, *nonvanishing* 3OD and negative 4OD was presented, its stability proven, and its conditions of existence clarified^12–15^. For this case, an exact $\text{sech}\times \text{tanh}$–shaped “dipole-soliton” solution was derived lately^16^.

Besides such single-pulse solitary wave solutions, various types of molecule-like bound states have been reported that consist of multiple pulses. This includes bound states consisting of two identical optical pulses separated by a fixed time-delay, realized through dispersion engineering for a standard NSE^17^, bound solitons arising in models of coupled NSEs^18–25^, bound solitons copropagating in twin-core fibers subject to higher-order dispersion^26^, and dissipative optical soliton molecule generated in passively mode-locked fiber laser^27,28^. More recently, a different kind of molecule-like bound state was reported that forms a single complex, consisting of two subpulses with roughly similar amplitudes but distinctly different center frequencies^29^. Such compound states are enabled by a propagation constant that allows for group-velocity matched copropagation of pulses in distinct domains of anomalous dispersion, separated by an interjacent domain of normal dispersion. A mutual cross-phase modulation induced attractive potential provides the binding mechanism that holds the constituent pulses together^29^. This transfers the concept of a soliton induced strong refractive index barrier for a normally dispersive wave^30^, to the interaction of pulses in distinct domains of anomalous dispersion. The former process is enabled by a general wave reflection mechanism originally reported in fluid dynamics^31^, in optics referred to as the push-broom effect^32^, optical event horizon^33,34^, or temporal reflection^35^, allowing for a strong and efficient all optical control of light pulses^36,37^. This mechanism has been shown to naturally appear in the supercontinuum generation process^38–41^. The previously studied formation of molecule-like two-frequency pulse compounds constitutes a paradigmatic example of extreme states of light, also offering intriguing insights to atom-like features of a soliton, including its ability to act as a localized trapping potential with a discrete level spectrum^29^. For a higher-order nonlinear Schrödinger equation with positive 2OD and negative 4OD, similar compound states where recently also observed, and, along with the $\text{sech}^2$–shaped single soliton solutions of earlier studies^9,12^, identified as members of a large family of generalized dispersion Kerr solitons^42^. Objects of this type have recently been observed within a mode-locked laser cavity^43^. Dual-frequency pulses with similar pulse structure have previously also been studied experimentally in passively mode-locked fiber lasers^44^, and in a model for dual-channel simultaneous modelocking based on the Swift-Hohenberg equation^45^. Further, two-color soliton microcomb states where reported in theoretical studies of Kerr microresonators in terms of the Lugiato-Lefever equation (LLE) with two separate domains of anomalous dispersion^46^, and in the standard LLE with added negative quartic group-velocity dispersion^47^. Bound states of distinct solitons, i.e. composite solitons, with a very similar pulse structure where reported in a combined theoretical and experimental study of the Kerr multistability in the LLE^48^. The properties of these kind of objects, which are referred to by a variety of names such as dual-frequency pulses^44^, two-color soliton states^46^, two-frequency soliton molecules^29^, composite solitons^48^, and, polychromatic soliton molecules^43^, are largely unexplored. Subsequently we refer to these objects simply as pulse compounds.

Here, we study the interaction dynamics of two initially superimposed fundamental solitons at distinctly different center frequencies in terms of a propagation constant for which the group velocity dispersion (GVD) has downward parabolic symmetry. Such a profile allows to parametrically define pairs of center frequencies at which the local dispersion parameters have the same absolute values at any order. This reduces the complexity of the underlying model and allows to explore the influence of the nonlinear interaction on the model dynamics more directly. Specifically, we here investigate how an initial group-velocity (GV) mismatch affects the formation of two-frequency pulse compounds. While it was shown that such compound states can compensate sufficiently small GV mismatches through excitation of internal degrees of freedom^29^, reminiscent of molecular vibrations, this puts their robustness to the test and sheds more light on the binding mechanism that holds the subpulses together. In the limit of large GV mismatch we observe a crossover from the formation of two-frequency compound states to escaping solitons. We demonstrate that the crossover region exhibits pulse compounds consisting of a strong trapping pulse and a weak trapped pulse, GV matched despite a large center frequency mismatch. Building upon the interaction of a single soliton with a localized attractive potential in terms of a perturbed NSE, we derive a simplified theoretical approach that suggests an analogy to classical mechanics and allows to accurately estimate the parameter range wherein pulse compounds are formed.

## Results

**Figure 1:**
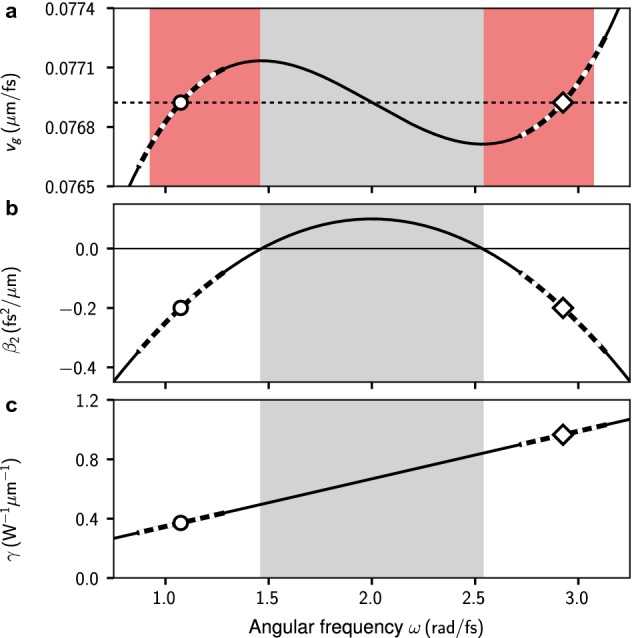
Specifics of the considered *z*-propagation model. (**a**) Frequency dependence of the group velocity. Frequency ranges shaded in red allow for group-velocity matched co-propagation of light pulses in separate regions of anomalous dispersion. Horizontal dashed line indicates reference velocity $v_0$. (**b**) Group velocity dispersion profile. (**c**) Nonlinear coefficient function. In all subplots, normally dispersive frequency ranges are shaded gray. Open circle and open square indicate the loci of $\omega _{\text{GVM}1}$ and $\omega _{\text{GVM}2}$, respectively. Thicker dashed parts of curves indicate the angular frequency ranges covered by the parameter sweep. Polynomial models for $\beta (\omega )$ and $\gamma (\omega )$ are detailed in the main text.

We model *z*-propagation of the real-valued optical field $E(z,t)={\sum _\omega }\,E_{\omega }(z)e^{-i\omega t}$ in a periodic *t*-domain of extend *T* with $\omega \in \frac{2\pi }{T} \mathbb {Z}$ in terms of the complex-valued analytic signal $\mathcal {E}(z,t)=2\,{\sum _{\omega >0}}\, E_{\omega }(z)e^{-i\omega t}$ via the first-order nonlinear propagation equation

1 $$\begin{aligned} i\partial _{z}\mathcal {E}_{\omega } + \beta (\omega )\mathcal {E}_{\omega } + \gamma (\omega )\left( |\mathcal {E}|^2\mathcal {E}\right) _{\omega >0}=0, \end{aligned}$$

describing single mode propagation in a nonlinear waveguide^49,50^. In Eq. (1), $\beta (\omega )$ denotes the propagation constant and $\gamma (\omega )$ specifies a coefficient function for its nonlinear part. The characteristics of both are illustrated in Fig. 1. Considering the reference frequency $\omega _0=2\,\text {rad}/\text {fs}$, the propagation constant is modeled by the polynomial expression

2 $$\begin{aligned} \beta (\omega )=\sum _{n=0}^{4} \frac{\beta _n}{n!}\,\left( \omega -\omega _0\right) ^n, \end{aligned}$$

with $\beta _0 = 25.0 \,{\upmu \text {m}^{-1}}$, $\beta _1 = 13.0 \,{\text {fs}\,\upmu \text {m}^{-1}}$, $\beta _2 = 0.1\,{\text {fs}^2\,\upmu \text {m}^{-1}}$, $\beta _3 = 0.0\,{\text {fs}^3\,\upmu \text {m}^{-1}}$, and $\beta _4 = -0.7\,{\text {fs}^4\,\upmu \text {m}^{-1}}$. For our subsequent numerical analysis we consider the transformed field $\mathcal {E}^\prime _\omega (z)=\mathcal {E}_\omega (z)\exp (i\frac{\omega }{v_0}z)$, shifted to a moving frame of reference. The time-domain representation $\mathcal {E}^\prime (z,t)$ then corresponds to the time-shifted analytic signal $\mathcal {E}(z,\tau =t-z/v_0)$. The reference velocity $v_0$ is chosen so that the time-domain dynamics appears slow. We subsequently set $v_0 \equiv v_g(\omega _0)\approx 0.0769\,{\upmu \text {m}/\text {fs}}$, wherein $v_g(\omega )\equiv [\partial _\omega \beta (\omega )]^{-1}$ signifies the group-velocity, see Fig. 1a. As can be seen in Fig. 1b, the group velocity dispersion (GVD) $\beta _2(\omega )=\partial _\omega ^2 \beta (\omega )$ assumes a downward parabolic shape, which, in terms of the angular frequency detuning $\Omega =\omega -\omega _0$, can be expressed as $\beta _2(\omega _0+\Omega )=\beta _2 + \frac{\beta _4}{2}\Omega ^2$. It is thus similar to the setup considered in reference^42^ in which a NSE subject to positive quadratic and additional negative quartic dispersion was studied (see “Methods” for details). It is further a simplified variant of the propagation constant with a non-symmetric GVD, for which we previously studied the interaction of solitons leading to the formation of heteronuclear soliton molecules^29^. Here, the zero-dispersion detunings are given by the roots of the GVD at $\Omega _{{{\text{ZDW}1},{\text{ZDW}2}}}=\pm \sqrt{-2\beta _2/\beta _4} \approx \pm 0.535 \,\text {rad}/\text {fs}$, specifying two zero-dispersion frequencies at $(\omega _{\text{ZDW}1},\omega _{\text{ZDW}2})\approx (1.465, 2.535)\,\text {rad}/\text {fs}$. The coefficient function of the nonlinearity is modeled as

3 $$\begin{aligned} \gamma (\omega )=\gamma _0 + \gamma _1 \omega , \end{aligned}$$

with $\gamma _0=0.026\,{\text {W}^{-1} \upmu \text {m}^{-1}}$ and $\gamma _1=0.321\,{\text {fs}\, \text {W}^{-1} \upmu \text {m}^{-1}}$, see Fig. 1c. To better understand the time-frequency interrelations of the analytic signal at a selected propagation distance *z*, we consider its spectrogram^51^

4 $$\begin{aligned} P_{S}(\tau ,\omega ) = \frac{1}{2 \pi } \left| \int \mathcal {E}\left( z,\tau ^\prime \right) h\left( \tau ^\prime -\tau \right) e^{-i \omega \tau }~{\text{d}}\tau ^\prime \right| ^2, \end{aligned}$$

wherein $h(x)=\exp (-x^2/2\sigma ^2)$ specifies a Gaussian window function with root-mean-square width $\sigma$, used to localize $\mathcal {E}(z,\tau )$ in time.

Equation (1) is free from the slowly varying envelope approximation but can be reduced to the generalized nonlinear Schrödinger equation by introduction of a complex envelope for a suitable center frequency^49^. By assuming $\gamma =\text{const.}$, it can further be reduced to a standard NSE with higher orders of dispersion. For the propagation of an initial field in terms of Eq. (1) we use a pseudospectral scheme implementing *z*-propagation using a fourth-order Runge-Kutta method^52^.

### Initial conditions

As pointed out above, the GVD is symmetric about $\omega _0=2\,\text {rad}/\text {fs}$. Two frequencies are group-velocity (GV) matched to $\omega _0$. In terms of the angular frequency detuning they are located at $\Omega _{{{\text{GVM}1}, {\text{GVM}2}}} = \pm \sqrt{ -6 \beta _2/\beta _4}\approx \pm 0.926\,\text {rad}/\text {fs}$, specifying group-velocity matched frequencies at $(\omega _{\text{GVM}1}, \omega _{\text{GVM}2}) \approx (1.074, 2.926)\,\text {rad}/\text {fs}$, see Fig. 1a. Both frequencies are located in distinct domains of anomalous dispersion realized by the considered propagation constant, see Fig. 1b. In general, group-velocity matched co-propagation of anomalously dispersive light pulses is possible in the frequency ranges highlighted in red in Fig. 1a. More specifically, for the considered propagation constant, a mode in range $\omega \in (0.931\,\text {rad}/\text {fs},~\omega _{\text{ZDW}1})$ is GV matched to a mode in $\omega \in (\omega _{\text{ZDW}2},~3.069\,\text {rad}/\text {fs})$.

Subsequently we will consider two fundamental solitons with duration $t_0=20\,\text {fs}$ at distinctly different center frequencies $\omega _1=\omega _{\text{GVM}1}-\Delta \omega$ and $\omega _2=\omega _{\text{GVM}2}+\Delta \omega$, with frequency offset parameter $\Delta \omega \in (-0.2,0.2)\,\text {rad}/\text {fs}$. A parameter sweep over these values of $\Delta \omega$ covers the frequency ranges highlighted by the thickened dashed curves in Fig. 1. A full initial condition for the real-valued optical field reads

5 $$\begin{aligned} E(0,t) = \mathsf {Re}\left[ A_1\,e^{-i\omega _1 t} {\text{sech}}\left( t/t_0\right) + A_2\,e^{-i\omega _2 t} {\text{sech}}\left( t/t_0\right) \right] . \end{aligned}$$

The initial pulses are specified by the amplitude condition for a fundamental soliton, given by $A_{1,2}=\sqrt{|\beta _2(\omega _{1,2})|/\gamma (\omega _{1,2})}/t_0$. Thus, for any considered value of $\Delta \omega$, both initial solitons will have matching dispersion lengths, i.e. $L_{D,1}=L_{D,2}$ with $L_{D,1}=t_0^2/|\beta _2(\omega _{1})|$ and $L_{D,2}=t_0^2/|\beta _2(\omega _{2})|$. However, since $A_1 = \sqrt{\gamma (\omega _2)/\gamma (\omega _1)} A_2$, their amplitudes satisfy $A_1>A_2$. The group-velocity mismatch of both solitons vanishes only at $\Delta \omega =0$ and increases for increasing absolute values of $\Delta \omega$. For example, for $\Delta \omega < 0$ one has $v_g(\omega _1)\ge v_0 \ge v_g(\omega _2)$. In the considered frame of reference, a localized pulse with $v<v_0$ will move towards larger values of $\tau$ for increasing distance *z*.

The solitons injected at $\omega _1$ and $\omega _2$ are subject to higher orders of dispersion, which, in principle, causes their velocities to slightly deviate from their bare group-velocities $v_g(\omega _1)$ and $v_g(\omega _2)$, respectively^5,53^. For a soliton with center frequency $\omega _s$ and duration $t_s$, this might be taken into account by considering a “corrected” soliton velocity^54^ $v_g^\prime (\omega _s, t_s) = \left[ \beta _1(\omega _s) - \beta _2(\omega _s)/(\omega _s t_s^2) + \beta _3(\omega _s)/(6 t_s^2)\right] ^{-1}$. For the full range of simulation parameters considered in the presented study, the largest relative difference of these velocities was found to be $|v_g-v_g^\prime |/v_g < 10^{-4}$. Subsequently we opted to use the usual group-velocity $v_g$ when referring to the velocity of the initial solitons.

### Propagation dynamics of limiting cases

**Figure 2:**
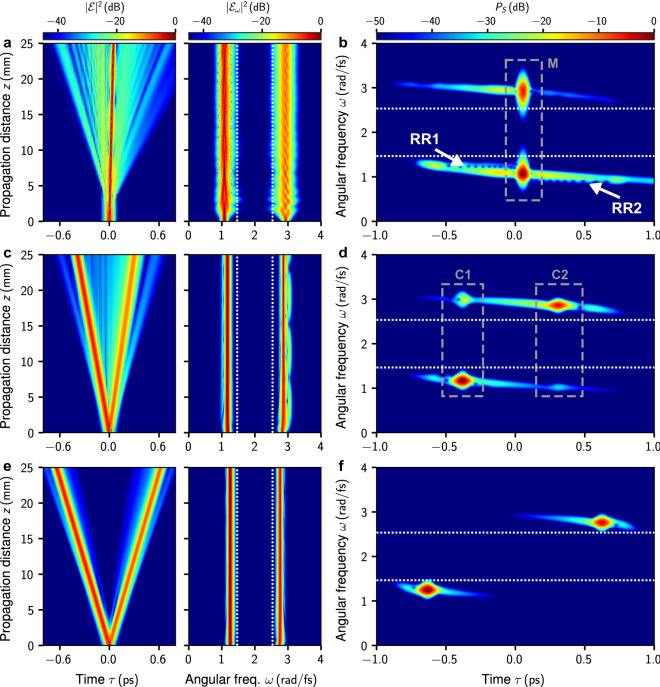
Exemplary propagation dynamics. (**a**) Evolution of the normalized time-domain intensity $|\mathcal {E}(z,t)|^2/{\text {max}}[|\mathcal {E}(z=0\,\text {mm},t)|^2]$ and normalized spectral intensity $|\mathcal {E}_\omega (z)|^2/{\text {max}}[|\mathcal {E}_\omega (z=0\,\text {mm})|^2]$ of the analytic signal for $\Delta \omega = 0\,\text {rad}/\text {fs}$. Vertical dashed lines indicate zero dispersion points. (**b**) Analytic signal spectrogram at $z=25\,\text {mm}$ for $\Delta \omega = 0\,\text {rad}/\text {fs}$. Horizontal dashed lines indicate zero dispersion points. Dashed box (labeled M) encloses a molecule-like compound state. Trains of nodes signaling generation of resonant radiation are labeled RR1 and RR2. (**c**,**d**) Same as (**a**,**b**) for $\Delta \omega = -0.1\,\text {rad}/\text {fs}$. In (**d**), the two dashed boxes (labeled C1 and C2) enclose pulse compounds each characterized by a strong trapping pulse and a weak trapped pulse. (**e**,**f**) same as (**a**,**b**) for $\Delta \omega = -0.18\,\text {rad}/\text {fs}$. Spectrograms are computed using $\sigma =20\,\text {fs}$ in Eq. (4).

Our earlier study of the interaction dynamics of initially overlapping group-velocity matched fundamental solitons with a vast frequency gap^29^, suggests that in the limiting case of group-velocity matched initial solitons [$\Delta \omega = 0\,\text {rad}/\text {fs}$], a heteronuclear two-frequency pulse compound will form. The evolution of a corresponding initial condition in the propagation range $z=0{-}25\,\text {mm}$ is shown in Fig. 2a. The composite pulse generated by this initial condition, highlighted in the spectrogram in Fig. 2b, consists of two subpulses with roughly similar amplitudes but distinctly different center frequencies. From the spectral intensity $|\mathcal {E}_\omega |^2$ and the spectrogram $P_S$, the vast frequency gap between both subpulses is clearly evident. It generates resonant radiation upon propagation and leads to a kind of “radiating” compound state. In Fig. 2b, these resonances are signaled by trains of nodes that separate from the localized state. A thorough analysis of a pulse compound with a similar composition was detailed in reference^29^ [see Fig. 2(f) of that reference]. The binding mechanism that leads to the formation of such a composite pulse is realized by the mutual cross-phase modulation between its interacting subpulses^29^. The resulting pulse compounds are quite robust: small initial group-velocity mismatches can be compensated by frequency shifts of the subpulse center frequencies. This enables intriguing internal dynamics, reminiscent of molecular vibrations, examined more closely in Fig. 4 below. In the limiting case of a large group-velocity mismatch of the initial solitons, i.e. for large absolute values of $\Delta \omega$, we expect that both pulses escape their mutual binding. This is demonstrated for $\Delta \omega =-0.17\,\text {rad}/\text {fs}$ in Fig. 2e,f. As evident from the time-domain propagation dynamics in Fig. 2e, two separate localized states with nonzero relative velocity can indeed be identified. They can be distinguished well in the spectrogram in Fig. 2f, indicating no notable trapping by either pulse.

A crossover from the formation of two-frequency soliton compounds to escaping solitons can be expected based on two arguments. First, consider the point of view of mutual trapping of each pulse by a cross-phase modulation induced attractive potential formed by the other pulse^29^. Then, a classical mechanics interpretation of the propagation scenario suggests the existence of an escape velocity, sufficient for a particle to escape its trapping potential. We explore this analogy in more detail below. Second, for offset frequencies $\Delta \omega > 0.143\,\text {rad}/\text {fs}$, i.e. $\omega _1 <0.931\,\text {rad}/\text {fs}$ and $\omega _2>2.926\,\text {rad}/\text {fs}$, no mode can be group-velocity matched to either initial soliton, see Fig. 1a. Having demonstrated the propagation dynamics for two specific values of the frequency offset parameter $\Delta \omega$, a thorough investigation of the crossover between the above limiting-cases in terms of $\Delta \omega$ is in order.

### Crossover from mutual trapping to escape

**Figure 3:**
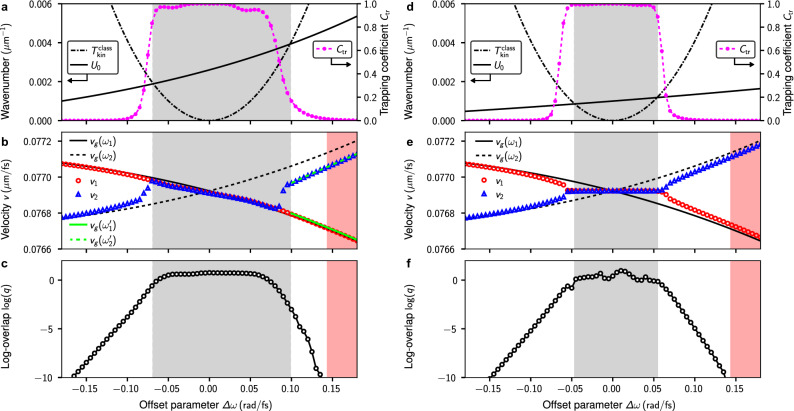
Characterization of the crossover from mutual trapping to escape. (**a**–**c**) Results for $\gamma (\omega )$ given by Eq. (3). (**a**) Point particle motion in an attractive potential. The particle can escape the well if its kinetic energy $T_{\text{kin}}^{\text{class}}$ exceeds the potential depth $U_0$ (see text for details). Parameter range in which the particle cannot escape the well is shaded gray. Secondary ordinate shows the trapping coefficient $C_{\text{tr}}$ computed in a simplified model for a soliton interacting with a localized attractive potential (see text for details). (**b**) Comparison of observed asymptotic velocities $v_1$ and $v_2$ of the dominant localized pulses in the distinct domains of anomalous dispersion and corresponding propagation constant based group-velocities $v_g$. Light-green solid and dashed lines indicate the group velocities $v_g(\omega _1^\prime )$ and $v_g(\omega _2^{\prime })$, obtained for the shifted pulse center frequencies $\omega _1^\prime$ and $\omega _2^\prime$, respectively (see text for details). (**c**) Logarithm of the overlap parameter *q* at $z=25\,\text {mm}$, quantifying the degree of mutual trapping (see text for details). Shaded area beyond $\Delta \omega \approx 0.143\,\text {rad}/\text {fs}$ indicates region in which group-velocity matching cannot be achieved, cf. Fig. 1a. (**d**–**f**) Same as (**a**–**c**) considering $\gamma (\omega )=\gamma _0$.

To better characterize the crossover from mutual trapping to unhindered escape of the initial solitons, we track the velocities of the dominant localized pulses in each domain of anomalous dispersion. In Fig. 3b, the asymptotic velocities associated with the initial solitons at $\omega _1$ and $\omega _2$ are labeled $v_1$ and $v_2$, respectively. In relation to the two limiting cases illustrated earlier, we find that at $\Delta \omega =0\,\text {rad}/\text {fs}$ (cf. Fig. 2a) the velocities of the compounds subpulses match each other and are in good agreement with the group-velocities of the initial solitons. At $\Delta \omega = -0.17\,\text {rad}/\text {fs}$ (cf. Fig. 2e) we find that the dominant pulses in each region of anomalous dispersion are clearly distinct, again in agreement with the group-velocities of the initial solitons. In between, a sudden crossover occurs at $\Delta \omega _c^{(-)} \approx -0.075\,\text {rad}/\text {fs}$, where $v_2$ shifts from $v_2=v_1\lesssim v_g(\omega _1)$ [for $\Delta \omega _c^{(-)}< \Delta \omega <0$] to $v_2 = v_g(\omega _2)$ [for $\Delta \omega < \Delta \omega _c^{(-)}$], see Fig. 3b.

**Figure 4:**
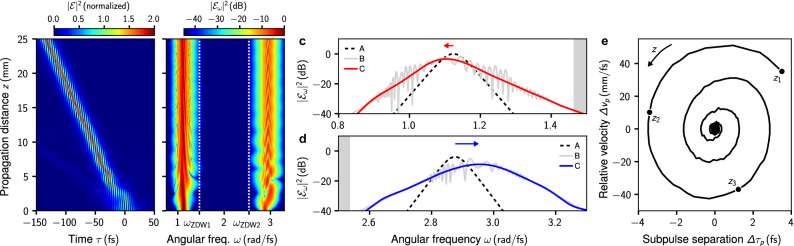
Formation of a two-frequency pulse compound at $\Delta \omega = -0.05\,\text {rad}/\text {fs}$. Evolution of (**a**) normalized time-domain intensity $|\mathcal {E}(z,t)|^2/{\text {max}}[|\mathcal {E}(z=0\,\text {mm},t)|^2]$ (shown on linear scale), and (**b**) normalized spectrum $|\mathcal {E}_\omega (z)|^2/{\text {max}}[|\mathcal {E}_\omega (z=0\,\text {mm})|^2]$. (**c**) Spectrum in the frequency range $(0.8,1.5)\,\text {rad}/\text {fs}$, showing the initial spectrum at $z=0\,\text {mm}$ (labeled A), the full spectrum at $z=25\,\text {mm}$ (labeled B), and a filtered spectrum at $z=25\,\text {mm}$ (labeled C), which excludes the free radiation and highlights the subpulse in the shown frequency range. Superimposed arrow indicates direction and size of observed frequency shift (numeric values are quoted in the text). (**d**) Same as (**c**) for frequency range $(2.5,3.3)\,\text {rad}/\text {fs}$. In (**c**,**d**) the domain of normal dispersion is shaded gray. (**e**) Internal dynamics of the pulse compound described in terms of separation ($\Delta t_p$) and relative velocity ($\Delta v_p$) of its subpulses. Trajectory in $(\Delta t_p,\Delta v_p)$-plane is shown for $z>4\,\text {mm}$. Markers indicate propagation distances $(z_1,z_2,z_3)=(4,5,6)\,\text {mm}$.

Matching subpulse velocities in the range $\Delta \omega _c^{(-)}< \Delta \omega <0$ result from an initial transient propagation regime during which the mutual interaction of the initially superimposed pulses causes both pulse center frequencies to shift, thereby also changing the pulse spectrum. In this parameter range we observe that the soliton with higher amplitude, i.e. the soliton initially at $\omega _1$, assumes a dominant role. While the effect on this pulse is small, the effect on the pulse initially at $\omega _2$ is rather large. This is shown in Fig. 4, where we detail a simulation run at $\Delta \omega = -0.05\,\text {rad}/\text {fs}$. An initial transient behavior in range $z<10\,\text {mm}$ is well visible, see Fig. 4a,b. In the latter, the initial velocity mismatch of both pulses induces a vivid dynamics. This is demonstrated in Fig. 4e, where the internal dynamics of the composite pulse in terms of the separation and relative-velocity of its subpulses, reminiscent of molecular vibrations, is shown. For this example we find the asymptotic frequency shifts $\omega _1 = 1.124\,\text {rad}/\text {fs}\rightarrow \omega _1^\prime \approx 1.113 \,\text {rad}/\text {fs}$ (Fig. 4c) and $\omega _2 = 2.876\,\text {rad}/\text {fs}\rightarrow \omega _2^\prime \approx 2.949 \,\text {rad}/\text {fs}$ (Fig. 4d). The frequency up-shift $\omega _2\rightarrow \omega _2^\prime$ is expected to result in a pulse velocity for which $v_g(\omega _2^\prime )>v_g(\omega _2)$ (cf. Fig. 1a). More precisely, we find the velocity shift $v_g(\omega _2)= 0.076868\,{\upmu \text {m}/\text {fs}} \rightarrow v_g(\omega _2^\prime )=0.07695\,{\upmu \text {m}/\text {fs}}$ in agreement with the data shown in Fig. 3b. As evident from Fig. 4a, radiation is emitted predominantly in the initial stage of the pulse compounds formation process.

**Figure 5:**
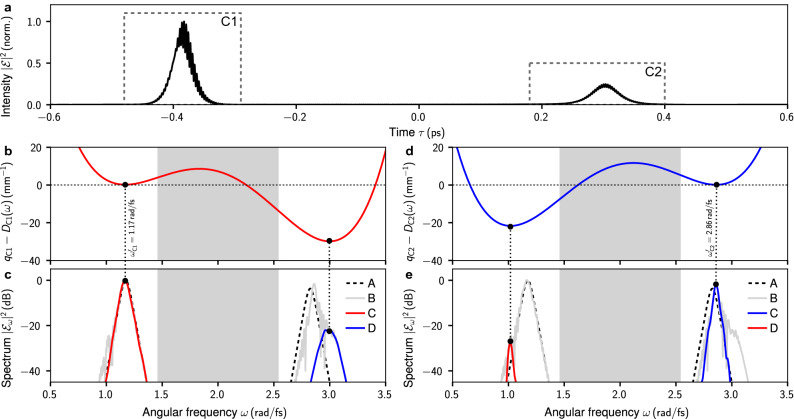
Detailed analysis of the pulse compounds C1 and C2 of Fig. 2d at $z=25\,\text {mm}$. (**a**) Normalized time-domain intensity $|\mathcal {E}|^2/{\text {max}}[|\mathcal {E}|^2]$. (**b**) Phase matching analysis for the strong trapping pulse of C1. Shift of the soliton wavenumber $q_{\text{C}1}=\gamma (\omega ^\prime _{\text{C}1}) P_0/2$ and wavenumber $D_{\text{C}1}(\omega ) = \beta (\omega ) - \beta (\omega ^\prime _{\text{C}1}) - \beta _1(\omega ^\prime _{\text{C}1})(\omega -\omega ^\prime _{\text{C}1})$, where $P_0$ is the peak intensity of the strong trapping pulse and $\omega ^\prime _{\text{C}1}$ is its center frequency. Local extrema indicate group velocity matching with the strong trapping pulse. Frequencies at which resonant radiation might be expected are indicated by the roots of $q_{\text{C}1}-D_{\text{C}1}(\omega )$. (**c**) Normalized spectrum $|\mathcal {E}_\omega (z)|^2/{\text {max}}[|\mathcal {E}_\omega (z=0\,\text {mm})|^2]$ showing the initial spectrum at $z=0\,\text {mm}$ (labeled A), full spectrum at $z=25\,\text {mm}$ (labeled B), and spectra of the strong trapping pulse (labeled C) and weak trapped pulse (labeled D) of C1. (**d**,**e**) Same as (**b**,**c**) for C2.

We find that in the vicinity of $\Delta \omega _c^{(-)}$, the asymptotic state is characterized by two distinct pulse compounds. The *z*-evolution of a corresponding initial condition at $\Delta \omega = -0.1\,\text {rad}/\text {fs}$ is shown in Fig. 2c,d. Therein, the time-domain propagation dynamics (left panel of Fig. 2c) shows two localized pulses that separate from each other for increasing propagation distance. As evident from the spectrogram at $z=25\,\text {mm}$ (Fig. 2d), the two localized pulses are actually pulse compounds (labeled C1 and C2 in Fig. 2d), each consisting of a strong trapping pulse and a weak trapped pulse. An analogous phenomenon, referred to as development of a “soliton shadow”, “mixing”, or “soliton-radiation trapping”, exists for coupled NSEs describing soliton propagation in birefringent fibers^21,25^, and gas-filled hollow-core photonic crystal fibers^55^. One of the main differences to other works is that we here allow for group velocity matching across a vast frequency gap, which plays an important role in observing this effect. For this reason, other studies of initially superimposed solitons with center frequency mismatch did not observe such an effect^56,57^. Figure 5 shows a more comprehensive analysis of the individual pulse compounds. As evident from Fig. 5a, the time-domain intensity of both pulse compounds exhibit a fringe pattern signaling the superposition of subpulses with a significant center frequency mismatch. In Fig. 5b,c (Fig. 5d,e), the spectrum of the compound labeled C1 [C2] is put under scrutiny. In either case, both subpulses are group velocity matched and a phase-matching analysis for the strong trapping pulse indicates no generation of resonant radiation^6,7^, see Fig. 5b,d. This is different from the radiating molecule in Fig. 2a.

For $\Delta \omega < \Delta \omega _c^{(-)}$, i.e. beyond the crossover region, the trapping phenomenon changes qualitatively. This can be seen from the overlap parameter

6 $$\begin{aligned} q(z) = \frac{\int I_1(z) I_2(z)~{\text{d}\tau }}{\int I_1(0)I_2(0)~{\text{d}\tau }}, \end{aligned}$$

in which $I_1(z)=|\sum _{\omega <\omega _{\text{ZDW}1}} \mathcal {E}_\omega (z) e^{-i\omega t}|^2$ and $I_2(z)=|\sum _{\omega >\omega _{\text{ZDW}2}} \mathcal {E}_\omega (z) e^{-i\omega t}|^2$ are the time-domain intensity profiles of the pulse components restricted to separate regions of anomalous dispersion. It quantifies the degree of mutual trapping and is shown in Fig. 3c. For the limiting case of the soliton molecule ($\Delta \omega = 0 \,\text {rad}/\text {fs}$; Fig. 2a) we find $q\approx 2.1$ at $z=25\,\text {mm}$. In the range $\Delta \omega < \Delta \omega _c^{(-)}$, i.e. progressing towards increasingly negative values of the frequency offset parameter, it shows an exponential decrease in support of a strong-trapping to weak-trapping transition. We find this also confirmed by comparing the spectrograms in Fig. 2d,f.

A similar crossover occurs for increasing positive values of the frequency offset parameter at $\Delta \omega _c^{(+)} \approx 0.08\,\text {rad}/\text {fs}$. For $\Delta \omega < \Delta \omega _c^{(+)}$ the values of $v_2$ and $v_1$ coincide and are again in well agreement with $v_g(\omega _1)$, see Fig. 3b. For $\Delta \omega < \Delta \omega _c^{(+)}$, $v_2$ crosses over to a value that follows the trend of $v_g(\omega _2)$, but exhibits the systematic deviation $v_g(\omega _2)-v_2 \approx 0.00007\,{\upmu \text {m}/\text {fs}}$. This systematic deviation is again a consequence of the perturbation imposed by the presence of a superimposed pulse in the initial condition. As pointed out earlier, the direct overlap of two solitons at $z=0\,\text {mm}$ leads to an initial transient stage, during which their mutual interaction causes both pulse center frequencies to shift. Here, the effect on the pulse initially at $\omega _1$ is again small and the effect on the pulse initially at $\omega _2$ is rather large. Analyzing the simulation run at $\Delta \omega = 0.12\,\text {rad}/\text {fs}$, we find the frequency shifts $\omega _1 = 0.954\,\text {rad}/\text {fs}\rightarrow \omega _1^\prime \approx 0.961 \,\text {rad}/\text {fs}$ and $\omega _2 = 3.046\,\text {rad}/\text {fs}\rightarrow \omega _2^\prime \approx 2.991 \,\text {rad}/\text {fs}$. The frequency down-shift $\omega _2\rightarrow \omega _2^\prime$ is expected to result in a pulse velocity for which $v_g(\omega _2^\prime )<v_g(\omega _2)$ (cf. Fig. 1a). As evident from Fig. 3b, the pulse velocities $v_g(\omega _1^\prime )$ and $v_g(\omega _2^{\prime })$ obtained for the shifted center frequencies are in excellent agreement with the observed pulse velocities (see light-green solid and dashed lines in Fig. 3b). As pointed out above, beyond $\Delta \omega =0.143\,\text {rad}/\text {fs}$, group velocity matching is not possible (shaded region in Fig. 3). This is reflected by the overlap parameter *q*, dropping down to negligible values for $\Delta \omega >0.143\,\text {rad}/\text {fs}$. We observe a shift of both pulse center frequencies towards each other for $\Delta \omega >0$, while they shift away from each other for $\Delta \omega <0$ (see the example detailed in Figs. 4c,d). This results in group-velocity matching in the domain where pulse compounds are formed, This is different from studies of the unperturbed NSE, where the center frequencies of initially overlapping solitons where reported to shift towards each other for any reasonable initial frequency separation^58^.

To clarify how the term $\propto \gamma _1 \omega$ in the definition of $\gamma (\omega )$ [Eq. (3)] affects our observations, we repeated the above parameter study using the modified coefficient function $\gamma (\omega )=\gamma _0$. This setting can be reduced to a standard NSE with higher orders of dispersion (see “Methods” for details), similar to the model in which generalized dispersion Kerr solitons were studied recently^42^. Considering this simplified coefficient function, the above parameter study involves two initial solitons with matching dispersion lengths [$L_{D,1}=L_{D,2}$] and equal amplitudes [$A_1=A_2$]. As shown in Fig. 3e, across the region of compound state formation (i.e. for $|\Delta \omega |<0.065\,\text {rad}/\text {fs}$), the asymptotic velocities $v_1$ and $v_2$ are not longer dominated by any particular pulse. Instead, the resulting composite pulse has velocity $v_0$. This is, again, achieved by a shift of the pulses center frequencies during an initial transient stage. In comparison to the case where $\gamma (\omega )$ is modeled via Eq. (3), we find that the region of compound state formation is narrower. Despite the higher orders of dispersion featured by Eq. (1), the results reported in Fig. 3e are in good qualitative agreement with the interaction dynamics of initially overlapping, group-velocity mismatched solitons in a model of two nonlinearly coupled NSEs^56^. Also, a systematically smaller value of the overlap parameter *q* is evident in Fig. 3f. Let us comment on the characteristics of the pulse compounds in the vicinity of the crossover. The distinct features of C1 and C2 in Fig. 5 are solely due to the unsymmetry caused by the coefficient function $\gamma (\omega )$ given by Eq. (3). Considering the above modified coefficient function, we find that the spectra of C1 and C2 are simply related by symmetry, i.e. we can obtain C2 by inversion of C1 about $\omega _0=2\,\text {rad}/\text {fs}$.

## Discussion

For the whole range of frequency offsets considered in our numerical simulations, we find that the observed velocity $v_1$ closely follows the group velocity $v_g(\omega _1)$. Both are associated with the initial fundamental soliton with the larger amplitude. We here find that the observed velocity $v_2$ can match $v_1$ in the range $-0.075\,\text {rad}/\text {fs}< \Delta \omega < 0.08\,\text {rad}/\text {fs}$, specifying the range within which heteronuclear pulse compounds are formed by the considered initial conditions. Outside this range, the formation of a single two-frequency soliton molecule is inhibited, with two localized pulses separating from each other and suppressed trapping for large absolute values of the frequency offset parameter.

We found that we can estimate the domain of molecule formation in terms of a simplified theoretical approach (see “Methods” for details). In the latter, the dynamics of a two-pulse initial condition of the form of Eq. (5), governed by the nonlinear propagation equation Eq. (1), is approximated by the dynamics of a single pulse evolving under a nonlinear Schrödinger equation with localized attractive potential, given by

7 $$\begin{aligned} i\partial _z \phi \left( z,\tau ^\prime \right) + \left[ i \beta _1^\prime \partial _{\tau ^\prime } - \frac{\beta _2^\prime }{2} \partial _{\tau ^\prime }^2 - U\left( \tau ^\prime \right) + \gamma ^\prime |\phi \left( z,\tau ^\prime \right) |^2 \right] \phi \left( z,\tau ^\prime \right) = 0. \end{aligned}$$

Therein the complex envelope $\phi (z,\tau ^\prime )$ describes the dynamics of the subpulse with smaller amplitude, i.e. the subpulse at $\omega _2$. The potential well $U(\tau ^\prime )$ is related to the subpulse with higher amplitude, i.e. the subpulse at $\omega _1$, and is given by $U(\tau ^\prime ) = - U_0\,{\text{sech}}^2(\tau ^\prime /t_0)$ with potential depth

8 $$\begin{aligned} U_0 = 2 \frac{ \gamma \left( \omega _2\right) }{ \gamma \left( \omega _1\right) } \frac{ |\beta _2\left( \omega _1\right) |}{t_0^2}. \end{aligned}$$

Further, $\beta _1^\prime = \beta _1(\omega _2)-\beta _1(\omega _1)$, $\beta _2^\prime = \beta _2(\omega _2)$, $\gamma ^\prime = \gamma (\omega _2)$, and $\tau ^\prime =t-\beta _1(\omega _1)z$. A similar approximation, for the special case of group-velocity matched propagation $\beta _1^\prime =0$, was recently used to demonstrate trapped states in a soliton-induced refractive index well^29^. Equation (7) suggests an analogy to a one-dimensional Schrödinger equation for a fictitious particle of mass $m=-1/\beta _2^\prime$, evolving in an attractive potential localized along the $\tau ^\prime$ axis. The relative velocity between the soliton and the potential is $\beta _1^\prime$. From a classical mechanics point of view we might expect that a particle, initially located at the potential center at $\tau ^\prime =0$, escapes the potential well if its “classical” kinetic energy along the $\tau ^\prime$-axis, given by

9 $$\begin{aligned} T_{\text{kin}}^{\text{class}}=\frac{m}{2} {\beta _1^\prime }^2 = -\frac{\left[ \beta _1\left( \omega _2\right) -\beta _1\left( \omega _1\right) \right] ^2}{2 \beta _2\left( \omega _2\right) }, \end{aligned}$$

exceeds the potential depth $U_0$. In other words, for $T_{\text{kin}}^{\text{class}}<U_0$ we expect the particle to remain trapped by the potential. For the original model, defined by Eq. (1), this might be used to approximately estimate the domain in which compound states are formed. The results of this simplified theoretical approach are summarized in Fig. 3a,d, where $T_{\text{kin}}^{\text{class}}$ and $U_0$ are shown as function of the frequency offset parameter $\Delta \omega$. For example, considering the setup with $\gamma (\omega )$ defined by Eq. (3), the condition $T_{\text{kin}}^{\text{class}}<U_0$ is satisfied for $-0.068\,\text {rad}/\text {fs}< \Delta \omega < 0.098\,\text {rad}/\text {fs}$ (Fig. 3a). Despite the various simplifying assumptions that led to the above trapping condition, the estimated bounds for the domain of compound state formation are in excellent agreement with the observed bounds discussed above. In Fig. 3a,d we complement the findings based on the classical mechanics analogy by probing the trapping-to-escape transition of a soliton in a potential well in terms of Eq. (20) via numerical simulations. We therefore computed a trapping coefficient, defined by

10 $$\begin{aligned} C_{\text{tr}} = \frac{1}{N} \int _{-10\,t_0}^{10\,t_0} |\phi \left( z,\tau ^\prime \right) |^2~{\text{d}}\tau ^\prime , \end{aligned}$$

with $N=\int |\phi (0,\tau ^\prime )|^2~{\text{d}}\tau ^\prime$ for $z=10\,\text {mm}$. Both are in excellent qualitative agreement.

In conclusion, we showed that there exists a limit in the group-velocity mismatch of the constituents of a solitonic two-frequency pulse compound, above which its existence is not possible anymore. We clarified the breakup dynamics for the compound states beyond that limit, and showed that every constituent takes away parts of the radiation, again depending on the relative group velocities. The velocity of the pulse compound before the breakup is determined mostly by its “heaviest” component. More generally, our work demonstrates clearly the limits of stability of multicolor solitonic pulse compounds and we expect that the presented crossover-phenomenon will be useful for studying and understanding the break-up dynamics of more complex multi-frequency compounds, such as the recently demonstrated polychromatic soliton molecules^43^.

## Methods

Below we derive a simplified theoretical model, allowing to estimate the parameter range of $\Delta \omega$ that supports formation of two-frequency pulse compounds discussed in the main text. Starting point of our consideration is the first order nonlinear propagation equation for the analytic signal [Eq. (1)], with propagation constant $\beta (\omega )$ and coefficient function $\gamma (\omega )$ given by Eqs. (2) and (3), respectively, together with initial conditions of the form of Eq. (5). We then make the following assumptions and approximation steps:

1. Introducing a reference frequency and shifting to a moving frame of reference^49^. We choose the reference frequency $\omega _0$, for which $\beta (\omega _0)=\beta _0$ and $\beta _1(\omega _0)=\beta _1$, and consider the frequency detuning $\Omega = \omega -\omega _0$ to define the complex envelope
  11 $$\begin{aligned} \psi (z,\tau ) = \sum _\Omega \psi _\Omega (z) e^{-i \Omega \tau },\quad \text {with}\quad \psi _\Omega (z) = \mathcal {E}_{\omega _0+\Omega }(z) e^{-i\left( \beta _0 + \beta _1 \Omega \right) z},\quad \text {and}\quad \tau =t-\beta _1 z, \end{aligned}$$
  for which Eq. (1) takes the form
  12 $$\begin{aligned} i\partial _z \psi _\Omega + \left[ \beta (\omega _0+\Omega ) - \beta _0 - \beta _1 \Omega \right] \psi _\Omega + \gamma (\omega _0+\Omega )\left( |\psi |^2 \psi \right) _{\Omega } = 0. \end{aligned}$$
  The initial condition Eq. (5) then reads
  13 $$\begin{aligned} \psi (0,\tau ) = \left( A_1 e^{-i\Omega _1 \tau } + A_2 e^{-i\Omega _2 \tau } \right) {\text{sech}}\left( \tau /t_0\right) , \end{aligned}$$
  with $\Omega _{1,2} = \omega _{1,2}-\omega _0$ and $A_{1,2}=\sqrt{|\beta _2(\omega _0+\Omega _{1,2})|/\gamma (\omega _0+\Omega _{1,2})}/t_0$. Let us note that, considering $\beta (\omega _0+\Omega )$ given by Eq. (2), and $\gamma (\omega _0+\Omega )=\gamma _0$, allows to simplify Eq. (12) to the higher-order nonlinear Schrödinger equation
  14 $$\begin{aligned} i\partial _z \psi _\Omega + \left( \frac{\beta _2}{2} \Omega ^2 + \frac{\beta _4}{24} \Omega ^4 \right) \psi _\Omega + \gamma _0\left( |\psi |^2 \psi \right) _\Omega = 0. \end{aligned}$$
  For parameter values $\beta _2>0$ and $\beta _4<0$, as we do consider here [see parameters listed right after Eq. (2)], Eq. (14) specifies the frequency domain representation of the model in which generalized dispersion Kerr solitons where demonstrated^42^.
2. Approximating the dynamics of a two-pulse initial condition [Eq. (13)], governed by Eq. (12), by a system of coupled higher-order nonlinear Schrödinger equations. Therefore, we define two distinct fields
  15 $$\begin{aligned} \chi ^{(1,2)}(z,\tau ) = \sum _{\varpi } \chi _\varpi ^{(1,2)}(z) e^{-i\varpi \tau }, \end{aligned}$$
  taken at the center frequencies $\Omega _{1,2}$ of the two pulses with $\varpi$ denoting the respective frequency detuning, and consider instead of Eq. (12) the pair of coupled equations
  16 $$\begin{aligned} i\partial _z \chi _\varpi ^{(1)} + \left( \beta _0^{(1)} + \beta _1^{(1)}\varpi + \frac{\beta _2^{(1)}}{2} \varpi ^2 + \frac{\beta _3^{(1)}}{6} \varpi ^3 + \frac{\beta _4^{(1)}}{24} \varpi ^4 \right) \chi _\varpi ^{(1)} + \gamma ^{(1)}\left( |\chi ^{(1)}|^2 \chi ^{(1)} + 2|\chi ^{(2)}|^2 \chi ^{(1)} \right) _\varpi = 0, \end{aligned}$$
  17 $$\begin{aligned} i\partial _z \chi _\varpi ^{(2)} + \left( \beta _0^{(2)} + \beta _1^{(2)}\varpi + \frac{\beta _2^{(2)}}{2} \varpi ^2 + \frac{\beta _3^{(2)}}{6} \varpi ^3 + \frac{\beta _4^{(2)}}{24} \varpi ^4 \right) \chi _\varpi ^{(2)} + \gamma ^{(2)}\left( |\chi ^{(2)}|^2 \chi ^{(2)} + 2|\chi ^{(1)}|^2 \chi ^{(2)} \right) _\varpi = 0. \end{aligned}$$
  In the linear parts of Eqs. (16, 17) we introduced the modified dispersion parameters $\beta _n^{(1,2)}\equiv \partial _\Omega ^n \left[ \beta (\omega _0+\Omega )-\beta _0 - \beta _1 \Omega \right] _{\Omega =\Omega _{1,2}}$, local to the center frequencies of both pulses. In the nonlinear parts of Eqs. (16, 17) we made the simplifying assumptions $\gamma ^{(1,2)}=\gamma (\omega _0+\Omega _{1,2})$ and kept only the effects of self-phase modulation and mutual cross-phase modulation. We might then approximate the dynamics of an initial condition of the form of Eq. (13), evolving under the single equation Eq. (12), by the pair of coupled equations Eqs. (16, 17) with initial conditions
  18 $$\begin{aligned} \chi ^{(1,2)}(0,\tau ) = A_{1,2} {\text{sech}}(\tau /t_0), \quad \text {where}\quad A_{1,2}= \sqrt{|\beta _2^{(1,2)}| / \gamma ^{(1,2)}}/t_0. \end{aligned}$$
  Thereby we further assume the spectral width of either pulse to be small compared to the separation of the pulses center frequencies. Let us note that in the special case where we use Eq. (12) to simulate the dynamics of a single pulse initial condition $\psi (0,\tau )=A_1e^{-i\Omega _1 \tau } {\text{sech}}(\tau /t_0)$ we can write $\chi ^{(1)}(z,\tau )=\sum _\varpi \psi _{\Omega _1+\varpi }e^{-i\varpi \tau }=\psi (z,\tau ) e^{i\Omega _1\tau }$, so that $\chi (0,\tau )=A_1 {\text{sech}}(\tau /t_0)$ follows immediately.
3. We make the simplifying assumption that $\beta ^{(1,2)}_n=0$ for $n>2$ in Eqs. (16, 17) and neglect the cross-phase modulation contribution in Eq. (16). The latter implies that the dynamics of $\chi ^{(1)}$, which represents the pulse with larger amplitude, is not affected by $\chi ^{(2)}$. Under these assumptions, Eq. (16) with $\chi ^{(1)}(0,\tau )=A_1{\text{sech}}(\tau /t_0)$ constitutes a standard nonlinear Schrödinger equation for a fundamental soliton. We may further use the transformation
  19 $$\begin{aligned} \phi (z,\tau ^\prime ) = \sum _\varpi \phi _\varpi e^{-i\varpi \tau ^\prime },\quad \text {with}\quad \phi _\varpi (z) = \chi ^{(2)}_\varpi (z) e^{-i\left( \beta _0^{(2)} + \beta _1^{(1)} \varpi \right) z}, \quad \text {and}\quad \tau ^\prime = \tau - \beta _1^{(1)} z, \end{aligned}$$
  to formally remove the term $\propto \beta _0^{(2)}$ from Eq. (17) and shift to a reference frame in which $|\chi ^{(2)}|^2$ is stationary. Abbreviating $\Delta \beta _1 \equiv \beta _1^{(2)}-\beta _1^{(1)}$ and introducing the potential $U(\tau ^\prime )\equiv -2\gamma ^{(2)}|\chi ^{(1)}(0,\tau ^\prime )|^2= - 2 \gamma ^{(2)} A_1^2 {\text{sech}}^2(\tau ^\prime /t_0)$ we obtain
  20 $$\begin{aligned} i\partial _z \phi _\varpi + \left( \Delta \beta _1 \varpi + \frac{\beta _2^{(2)}}{2} \varpi ^2 \right) \phi _\varpi + \left( \gamma ^{(2)}|\phi |^2 \phi - U \phi \right) _\varpi = 0. \end{aligned}$$
  We further consider the single soliton initial condition $\phi (0,\tau ^\prime )=A_2 {\text{sech}}(\tau ^\prime /t_0)$ [cf. Eq. (18)], initially localized at the center of the potential well. Let us note that in Eq. (19) $\tau ^\prime =t - \left( \beta _1 + \beta _2 \Omega _1 + \frac{\beta _4}{6} \Omega _1^3 \right) z = t - \beta _1(\omega _0+\Omega _1)z$, which verifies that Eq. (20) is in a reference frame in which the potential, representing the pulse at $\omega _1=\omega _0 + \Omega _1$, is at rest.

The time-domain representation of Eq. (20), given by

21 $$\begin{aligned} i\partial _z \phi (z,\tau ^\prime ) + \left[ i \Delta \beta _1 \partial _{\tau ^\prime } - \frac{\beta _2^{(2)}}{2} \partial _{\tau ^\prime }^2 - U(\tau ^\prime ) + \gamma ^{(2)}|\phi (z,\tau ^\prime )|^2 \right] \phi (z,\tau ^\prime ) = 0, \end{aligned}$$

constitutes the simplified model which allows to estimate the parameter range in which two-frequency pulse compounds are formed (see Discussion in the main text). Let us note that Eq. (21) represents a nonlinear Schrödinger equation with an attractive external potential of ${\text{sech}}$-squared shape. Similar model equations were previously used to study soliton-defect collisions in the nonlinear Schrödinger equation^59,60^, and interaction of matter-wave solitons with quantum wells in the one-dimensional Gross-Pitaevskii equation^61^. While these studies considered the collision of a soliton with an external attractive potential, our aim is here to understand the escape of a soliton from such a potential.
